# Longitudinal Study of SARS-CoV-2 Vaccinations and Infections in Patients with Gastrointestinal Cancer: Stabilizing Immune Responses and Neutralizing Emerging Variants with Variant-Adapted Antigen Exposures †

**DOI:** 10.3390/ijms252413613

**Published:** 2024-12-19

**Authors:** Maria A. Gonzalez-Carmona, Alina M. Schmitz, Moritz Berger, Leona I. Baier, Jens G. Gorny, Farsaneh Sadeghlar, Thomas Anhalt, Xin Zhou, Taotao Zhou, Robert Mahn, Christian Möhring, Thomas Linnemann, Matthias Schmid, Christian P. Strassburg, Christoph Boesecke, Jürgen K. Rockstroh, Anna-Maria Eis-Hübinger, Malte B. Monin

**Affiliations:** 1 Department of Internal Medicine I, University Hospital Bonn, Venusberg-Campus 1, 53127 Bonn, Germany; 2 Centre for Integrated Oncology (CIO), Aachen, Bonn, Cologne, Düsseldorf (ABCD), Partner-Site Bonn, Venusberg-Campus 1, 53127 Bonn, Germany; 3 Institute for Medical Biometry, Informatics and Epidemiology, University Hospital Bonn, Venusberg-Campus 1, 53127 Bonn, Germany; 4 Institute of Experimental Haematology and Transfusion Medicine, University Hospital Bonn, Venusberg-Campus 1, 53127 Bonn, Germany; 5 German Centre for Infection Research (DZIF), Partner-Site Cologne-Bonn, Venusberg-Campus 1, 53127 Bonn, Germany; 6 Institute of Virology, University Hospital Bonn, Venusberg-Campus 1, 53127 Bonn, Germany; 7 Infektionsmedizinisches Centrum Hamburg (ICH), Glockengießerwall 1, 20095 Hamburg, Germany

**Keywords:** SARS-CoV-2, immune responses, gastrointestinal cancer, metastases, hepatocellular cancer, waning immunity, booster antigen contacts, omicron neutralization

## Abstract

This longitudinal study examined how active gastrointestinal (GI) cancer types affect immune responses to SARS-CoV-2, focusing on the ability to neutralize the Omicron variants. Patients with GI cancer (*n* = 168) were categorized into those with hepatocellular carcinoma, hepatic metastatic GI cancer, non-hepatic metastatic GI cancer, and two control groups of patients with and without underlying liver diseases. Humoral and cellular immune responses were evaluated before and after Omicron antigen exposures. In the pre-Omicron era, humoral SARS-CoV-2 immunity decreased after three antigen contacts without further antigen exposure. While Omicron neutralization was significantly lower than wildtype neutralization (*p* < 0.01), Omicron infections were yet mild to moderate. Additional Omicron exposures improved IgG levels (*p* < 0.01) and Omicron neutralization (*p* < 0.01). However, this effect was significantly less intense in patients with active GI cancer, particularly in patients with pancreaticobiliary neoplasms (PBN; *p* = 0.04), with underlying immunodeficiency (*p* = 0.05), and/or under conventional chemotherapy (*p* = 0.05). Pre-Omicron SARS-CoV-2 immunity prevented severe clinical courses of infections with Omicron variants in patients with GI cancer. However, in patients with PBN, with underlying immunodeficiency, and/or under conventional chemotherapy initial contacts with Omicron antigens triggered only reduced immune responses. Thus, subgroups could be identified for whom booster vaccinations are of special clinical significance.

## 1. Introduction

Coronavirus disease 2019 (COVID-19) is currently classified as less dangerous than at the beginning of the pandemic [1]. Newer variants of severe acute respiratory syndrome coronavirus type 2 (SARS-CoV-2), in particular the currently predominant Omicron variants, are more transmissible than the wildtype [2]. However, the course of the infections is usually milder, as the average immunity has increased significantly due to vaccinations and/or infections [3,4,5]. Additionally, antiviral drugs improved the clinical outcome of high-risk patients [6,7,8,9]. Nevertheless, the Omicron variants are more resistant to neutralization by vaccines targeting the wildtype [10,11], and thus, remain associated with significant morbidity and mortality worldwide [12]. Patients with immunodeficiency, especially those with hematological neoplasms, and presumably impaired immune responses to SARS-CoV-2 antigen exposures, are still at particular risk of severe courses of COVID-19 in the event of infection with an Omicron variant [13,14].

Differentiated data on immune responses in subgroups of patients with solid neoplasms are scarce, and recommendations are often based on a transfer of data from patients with hematological neoplasms [15,16,17,18]. However, immune responses appeared to be more effective in patients with solid neoplasms than in patients with hematological neoplasms [19]. In a longitudinal study, we previously demonstrated that immune responses in the subgroup of patients with active gastrointestinal (GI) cancer, especially those with hepatocellular carcinoma (HCC), were less effective than in patients in follow-up care with a past medical history of GI cancer after basic SARS-CoV-2 immunization [20]. Impaired immune responses were closely related to the malignant disease itself, chemotherapeutic treatment regimens, and/or additional immunodeficiency rather than to any underlying liver dysfunction [20]. Although the differences were largely mitigated by a first pre-Omicron booster antigen exposure, a decline in titers was observed over time [21,22], which was confirmed by another study in patients with GI cancer [23].

The focus of our study so far has been on the ability to neutralize wildtype SARS-CoV-2, without considering emerging variants, i.e., currently the Omicron variants. Recent studies have shown that there was a weaker cross-reactive B- and T-cell response against Omicron variants following vaccination against wildtype SARS-CoV-2 in both healthy controls and oncological patients [24,25,26,27,28,29,30]. However, there is still insufficient knowledge about the ability to neutralize the Omicron variants in patients with GI cancer, and recommendations of national and international medical societies for these patients are largely based on pre-Omicron data [15,16,17,18].

As part of our longitudinal cohort study, we present new data on long-term immune responses in patients with HCC and other GI cancers. We additionally analyzed the ability to neutralize the Omicron variants before their emergence and after a first Omicron antigen exposure by variant-matched vaccination and/or infections. Thereby, we studied the impact of the cancer type, of the type of oncological treatment, in particular chemotherapeutic treatment, of any underlying liver disease, and of underlying immunodeficiency.

## 2. Results

### 2.1. Patients’ Characteristics

A total of 168 patients were enrolled in this cohort study (Table 2). In total, 39 (23.2%) patients had HCC (group 1), 26 (15.5%) patients had hepatic metastatic GI cancer (group 2), and 44 (26.2%) patients had non-hepatic metastatic GI cancer (group 3). CRC and PBN were the most represented non-hepatocellular GI cancer types. The control group consisted of 59 (35.1%) patients in follow-up care for GI cancer without active cancerous disease and not under maintenance therapy. Of these control patients, 37 (62.7%) did not have underlying liver diseases (control 1), while 22 (37.3%) did (control 2). The most common liver diseases among them were metabolic dysfunction associated steatotic liver disease and alcoholic liver disease.

### 2.2. Pre-Omicron Immune Responses

**Figure 1:**
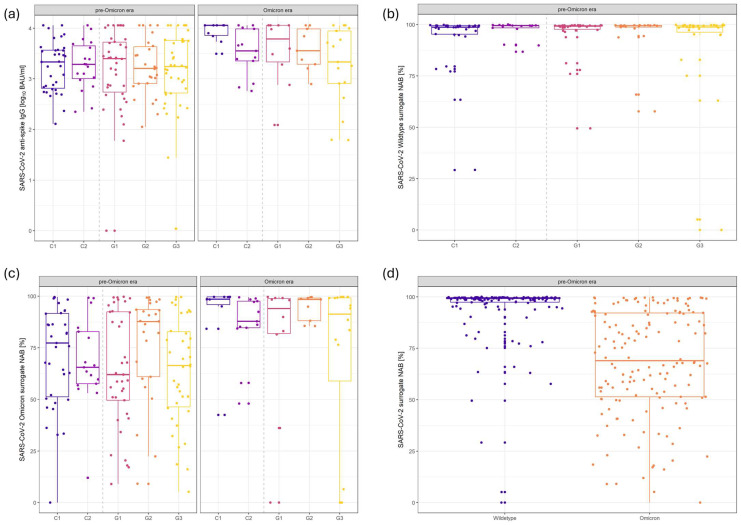
Humoral immune responses to pre-Omicron and Omicron SARS-CoV-2 antigen exposures. Balanced levels of SARS-CoV-2 anti-spike IgG (**a**) in all patients in the pre-Omicron era. Moreover, the capacities to neutralize the wildtype (**b**) as well as the Omicron variants (**c**) of SARS-CoV-2 were comparable in all patients. However, neutralization of the wildtype was significantly higher than that of the Omicron variants (**d**). Following Omicron antigen exposures, both levels of SARS-CoV-2 anti-spike IgG (**a**) as well as the capacity to neutralize the Omicron variants (**c**) increased significantly. Abbreviations: C1: control 1 (patients in follow-up care without underlying liver disease); C2: control 2 (patients in follow-up care with underlying liver disease); G1: group 1 (patients with hepatocellular carcinoma); G2: group 2 (patients with hepatic metastic gastrointestinal cancer); G3: group 3 (patients with non-hepatic metastic gastrointestinal cancer); IgG: immunoglobulin G; sNABs: surrogate neutralization anti-bodies.

By September 2022, all patients had comparable relative levels of SARS-CoV-2 anti-spike IgG after a mean of 3.45 (2–5) SARS-CoV-2 antigen exposures (vaccinations against the wildtype and/or infections with pre-Omicron variants), with no significant differences compared to patients in follow-up care without any underlying liver disease (control 1: 3.24 log_10_ BAU/mL; 95% CI: 3.02–3.46; Figure 1a; Table 1a). Additionally, the capacities to neutralize wildtype SARS-CoV-2 (control 1: 93.24%; 95% CI: 87.86–98.63; Figure 1b; Table 1a) and/or the Omicron variants (control 1: 71.48%; 95% CI: 62.78–80.18; Figure 1c; Table 1a) were balanced among all groups of patients. Yet, the capacity to neutralize the Omicron variant was significantly lower than that of the wildtype in all patients of our cohort regardless of cancer activity, cancer type, and/or any underlying liver disease (*p* < 0.01; Figure 1d). The assumed effective titer of SARS-CoV-2 anti-spike IgG of 847.0 BAU/mL was achieved by 68.35% (*n* = 108/158) of all patients (Table 3). A specific subgroup of patients who did not achieve this titer could not been identified. In 83.3% (*n* = 50/60) of cases, a qualitative cellular immune response was detectable with no significant differences between the subgroups (Table 3, Interferon-Gamma Releasing Assay).

### 2.3. Impact of an Omicron Antigen Exposure

Omicron antigen contacts occurred mainly through infections (*n* = 67; 70.5%) but also through variant-adapted vaccines (*n* = 28; 29.5%). The course of infections was predominantly mild, with 97.0% (*n* = 65/67) of cases remained in the outpatient setting. Only two patients required hospitalization, and none needed intensive care (score 4–5 on the WHO Clinical Progression Scale; Table 3). Following any additional Omicron antigen contact, total SARS-CoV-2 anti-spike IgG levels (control 1: 3.96 log_10_ BAU/mL; 95% CI: 3.58–4.34; *p* < 0.01; Figure 1a; Table 1a) and the capacity to neutralize Omicron (control 1: 93.86%; CI: 78.21–100.0; *p* < 0.01; Figure 1c; Table 1a) increased significantly until September 2023. There was neither a qualitative nor a quantitative difference in the humoral immune responses between patients who had undergone an infection and those who were vaccinated. Only patients with non-hepatic metastatic GI cancer had a significantly weaker booster response for SARS-CoV-2 anti-spike IgG levels (group 3: 3.26 log_10_ BAU/mL; 95% CI: 2.97–3.55; *p* = 0.01; Figure 1a; Table 1a) compared to patients in follow-up care without an underlying liver disease. They also achieved the assumed effective titer of SARS-CoV-2 anti-spike IgG of 847.0 BAU/mL less frequently (70.59%; *n* = 12/17) than all other patients (81.36%; *n* = 48/59; *p* = 0.08) in the Omicron era.

A significant booster effect of Omicron antigen contacts on the cellular immune response was not observed in our cohort of patients.

### 2.4. Impact of Cancer Types and Underlying Liver Diseases on Immune Responses

**Figure 2:**
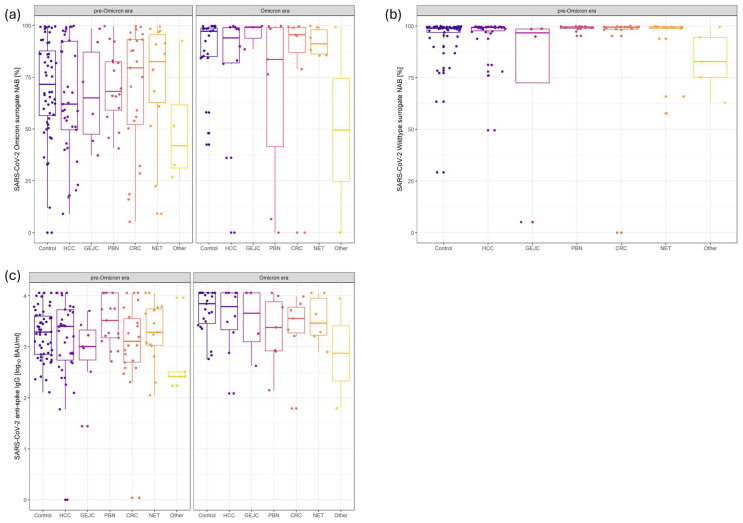
Impact of the type of cancer on humoral immune responses to pre-Omicron and Omicron SARS-CoV-2 antigen exposures. Balanced levels of SARS-CoV-2 anti-spike IgG (**a**) in all patients in the pre-Omicron era. Moreover, the capacity to neutralize the wildtype and/or the Omicron variants were comparable (**b**,**c**). Following Omicron antigen exposures, both levels of SARS-CoV-2 anti-spike IgG (**a**) as well as the capacity to neutralize the Omicron variants (**c**) increased significantly in all patients except for those with pancreaticobiliary cancer. “Other” included duodenal carcinoma, gastrointestinal stroma tumors, and cancer of unknown primary with most likely GI origin. Abbreviations: CRC: colorectal cancer; GEJC: gastroesophageal junction cancer HCC: hepatocellular carcinoma; IgG immunoglobulin; NET: neuroendocrine tumors; PBN: pancreaticobiliary neoplasms; sNABs: surrogate neutralization antibodies.

Patients with GEJC showed a significantly lower capacity to neutralize wildtype SARS-CoV-2 (74.30%; 95% CI 59.40–89.20; *p* = 0.01; Figure 2b; Table 1c) compared to liver-healthy patients without cancer activity (94.73%; 95% CI: 90.47–98.99) in the pre-Omicron era. Following an additional Omicron antigen contact, immune responses significantly improved in most patients with cancer activity including those with GEJC (Figure 2a,c; Table 1c). Only patients with PBN showed significantly reduced levels of SARS-CoV-2 anti-spike IgG (3.34 log_10_ BAU/mL; 95% CI: 2.89–3.79; *p* = 0.02; Figure 2a; Table 1c) as well as a significantly lower capacity to neutralize the Omicron variants (64.68%; 95% CI: 46.03–83.33; *p* = 0.02; Figure 2c; Table 1c) compared to liver-healthy patients without cancer activity (SARS-CoV-2 anti-spike IgG: 3.71 log_10_ BAU/mL; 95% CI: 3.46–3.96; Omicron sNAB: 90.96%; CI: 80.69–100.0).

Regarding different types of oncological treatment (chemotherapy, targeted therapy, immune-checkpoint inhibitors, local therapies, and combined therapies), no regimen was associated with a significantly impaired immune response in the pre-Omicron era (Table 1d). However, following an Omicron antigen contact, total levels of SARS-CoV-2 anti-spike IgG (*p* = 0.05) and the capacity to neutralize the Omicron variants (*p* = 0.05) were significantly lower in patients receiving conventional chemotherapy. This was not true for patients receiving targeted therapy, immune checkpoint inhibitors, local therapies, or combination therapies (Table 1d).

Compared to patients with underlying liver diseases (control 2), no differences concerning levels of SARS-CoV-2 anti-spike IgG (3.26 log_10_ BAU/mL; 95% CI: 2.95–3.57; Table 1b) or the capacities to neutralize the wildtype (97.52%; 95% CI: 89.98–100.0; Table 1b) and/or the Omicron variants of SARS-CoV-2 (67.84%; 95% CI: 55.12–80.55; Table 1b) were observed between the groups in the pre-Omicron era. Following an additional Omicron antigen exposure, a significantly increased capacity to neutralize the Omicron variants was revealed (control 2: 87.93%; 95% CI: 73.72–100.0; *p* = 0.01; Table 1b). In contrast, there was no booster effect on the levels of SARS-CoV-2 anti-spike IgG (control 2: 3.52 log10 BAU/mL; 95% CI: 3.16–3.88; *p* = 0.18; Table 1b).

### 2.5. Multivariable Analysis of Factors Influencing Immune Responses

**Figure 3:**
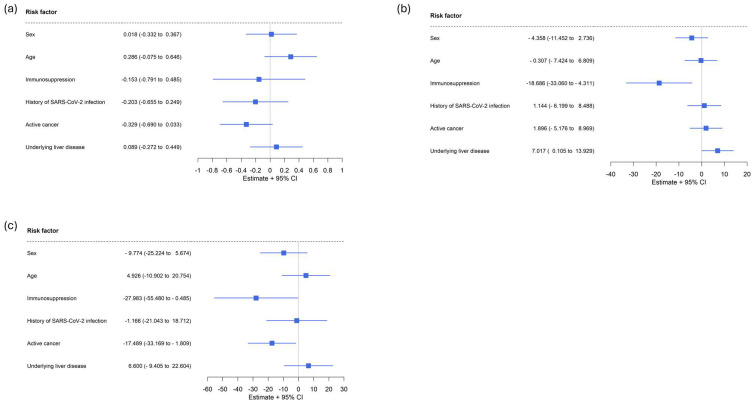
Multivariable analyses on factors potentially influencing immune responses to SARS-CoV-2 antigen exposures. (**a**) Effects on levels of SARS-CoV-2 anti-spike IgG. (**b**) Effects on the capacity to neutralize the wildtype of SARS-CoV-2. (**c**) Effects on the capacity to neutralize the Omicron variants of SARS-CoV-2. Any active cancerous disease under oncological treatment was associated with significantly lower SARS-CoV-2 anti-spike IgG levels (*p* = 0.04; (**a**)) and a lower capacity to neutralize the Omicron variants (*p* = 0.03; (**c**)). The ability to neutralize either the wildtype or the Omicron variants of SARS-CoV-2 was significantly impaired in patients with underlying relevant immunodeficiency (*p* = 0.01; (**b**) and *p* = 0.05; (**c**)). Considering different tumor types, it was confirmed that the capacity to neutralize the Omicron variant was significantly impaired in patients with PBN (*p* = 0.04).

After Omicron antigen contacts, any active cancerous disease under oncological treatment was associated with significantly lower SARS-CoV-2 anti-spike IgG levels (*p* = 0.04) and a lower capacity to neutralize Omicron variants (*p* = 0.03) in a multivariable analysis. In contrast, any underlying liver disease or any type of oncological treatment showed no significant effects on the immune response (Figure 3a,c). The ability to neutralize Omicron variants was additionally impaired in patients with underlying relevant immunodeficiency (*p* = 0.05; Figure 3c), which was also true for the ability to neutralize wildtype SARS-CoV-2 in the pre-Omicron era (*p* = 0.01; Figure 3b). Considering different cancer types, it was confirmed that the capacity to neutralize Omicron variants was significantly impaired in patients with PBN (*p* = 0.04).

## 3. Discussion

Patients with active GI cancer showed impaired immune responses after initial antigen contact with an Omicron variant of SARS-CoV-2 and especially a reduced ability to neutralize the Omicron variants. This was particularly pronounced in patients with systemic immunodeficiency and in patients with PBN (univariable and multivariable analysis) and to a lesser extent also in patients undergoing conventional chemotherapy (univariable analysis only).

Investigating longer-term immune responses and especially capacities to cross-reactively neutralize new variants of SARS-CoV-2 (i.e., Omicron in this case) in high-risk patients during the pre-Omicron era is crucial to understand their ability to maintain protective immunity and to evaluate their vulnerability to emerging variants. We found that humoral immune responses were lower than 24 weeks after the first pre-Omicron booster [22], but still balanced in September 2022 in all patients after a mean of 3.45 pre-Omicron antigen exposures. However, 93.24% neutralization of the wildtype was still ensured. Without any Omicron antigen contact having taken place before, a significantly lower cross-reactive neutralization of Omicron variants of 71.48% was observed for all patients of our cohort. Several studies have shown that Omicron neutralization improved after at least three pre-Omicron antigen contacts [31,32,33,34]. Nevertheless, cross-reactivity with Omicron variants after antigen contacts with pre-Omicron variants of SARS-CoV-2 were reported to be up to 8-fold lower [30]. Of note, in a cohort of cancer patients, of whom 58% suffered from solid cancer, a much better Omicron neutralization of 90% after three pre-Omicron antigen contacts was described [29], classifying patients with GI cancer as particularly vulnerable.

In a minority of only 16.7% of all study participants (*n* = 28/168), Omicron antigen exposures occurred through variant-adapted vaccination, as recommended by national and international guidelines for this patient group [15,16,17,18]. In line with the higher contagiousness compared to other variants of SARS-CoV-2 [2], most patients of our cohort were exposed to the Omicron variants through infections (39.9%; *n* = 67/168). In contrast, infections with pre-Omicron variants were significantly lower, at 5.1% in patients with active GI cancer and 2.4% in patients in follow-up care [22]. Consistent data from the pre-Omicron era indicated that overall clinical efficacy, with an 80–90% prevention rate of symptomatic courses of COVID-19 cases, can be assumed even in patients with hematological neoplasms [35]. Importantly, infections with the Omicron variants were generally mild in our cohort, with most patients being managed as outpatients, suggesting that exposures to pre-Omicron SARS-CoV-2 antigens provide substantial protection against infections with the Omicron variants from a clinical perspective. An efficient cellular immune response following an average of three exposures to pre-Omicron SARS-CoV-2 antigens was observed in over 83.3% of our patients, which likely contributed to the mild clinical outcomes. T-cell recognition appears to be relatively well-preserved against most SARS-CoV-2 variants, including Omicron, which is crucial for preventing severe COVID-19 [23,24,25,26,27].

After a continued steady decline in total levels of SARS-CoV-2 anti-spike IgG compared to our previous analyses [21,22], these levels increased significantly and were, thus, stabilized after an additional Omicron antigen exposure. Compared to the group of patients in follow up care with underlying liver disease (control 2), the titers were not stabilized by an Omicron antigen contact. This discrepancy may be attributed to an impaired liver function, since antibody titers in patients with cirrhosis have been shown to decrease more rapidly over time compared to healthy controls [36,37,38], suggesting a lower booster effect in this population.

Even more importantly, the capacity to specifically neutralize the Omicron variants increased significantly in all patients except those with non-hepatic metastatic GI cancer, particularly in patients with PBN. Interestingly, patients with GEJC showed significantly reduced neutralization of the wildtype prior to an Omicron antigen contact, which was not revealed in our previous studies [21,22]. Additionally, patients with underlying immunodeficiency were consistently less able to neutralize SARS-CoV-2 variants throughout the entire survey period as described for comparable patients with myelodysplastic syndromes and/or acute myeloid leukemia [39]. Following the basic vaccination against SARS-CoV-2, neutralization of the wildtype was also significantly reduced, especially in patients with PBN, as well as those with HCC and/or CRC [20]. Compensation by a third antigen exposure with pre-Omicron variants [21,22] was evidently not sustainable in the long term. Regarding various types of treatment, systemic conventional chemotherapy with cytostatic drugs was especially associated with a significantly poorer neutralization of the Omicron variants. Chemotherapy regimens are particularly used in patients with PBN and/or GEJC, suggesting an association. In patients with active GI cancer, those who received conventional chemotherapy appeared to respond less favorably to the vaccination and must, therefore, be considered particularly at risk. Among patients with solid tumors, especially older individuals and those undergoing chemotherapy showed a faster decline in humoral immune responses compared to cellular immune responses after vaccination against various pathogens, including SARS-CoV-2 [40,41,42]. This decline was particularly pronounced in patients with hematological neoplasms and those receiving immunosuppressive therapies [43,44], but also in patients with GI cancer [23].

The strength of our study is that, to the best of our knowledge, it is the only longitudinal, albeit monocentric, prospective study of SARS-CoV-2 vaccination in the subgroup of patients with GI cancer, covering the period from January 2021 to September 2023. Thereby, we provide short-term and long-term data on immune responses to different variants of SARS-CoV-2. While the data are robust, we experienced a relatively high loss to follow-up due to the high COVID-19-independent, cancer-associated mortality in the cohort. Additionally, patients who were under best supportive care and/or had decompensated liver cirrhosis were excluded a priori, which relativizes some observations, especially in patients with impaired liver function. Subgroup analyses of the different GI cancerous entities were not possible due to the small group sizes. Furthermore, we did not assess Omicron-specific cellular immune responses, as we assumed T-cell responses would be relatively stable over time regardless of the variant.

## 4. Patients and Methods

### 4.1. Study Design

**Figure 4:**
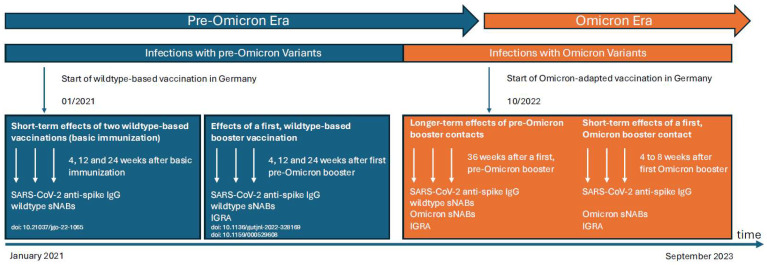
Study design. From the beginning of the second calendar week in 2022, Omicron was the predominant variant of SARS-CoV-2 in Germany, though the infection rate was initially very low in our cohort. Omicron-adapted vaccines became available in October 2022, and the number of Omicron infections significantly increased in our cohort at the same time. Based on this knowledge, we defined a pre-Omicron and an Omicron era. In three previous studies, we evaluated short-term effects of the basic immunization (4, 12, and 24 weeks after two wildtype-based vaccinations against SARS-CoV-2; first blue box), followed by analyses on the effects of a first wildtype-based booster vaccination (4, 12, and 24 weeks after a third wildtype-based booster vaccination; second blue box), as described elsewhere [19,20,21]. In the present analysis, we focused on the ability to neutralize the Omicron variants of SARS-CoV-2 (orange box). Thereby, we firstly investigated longer-term effects of pre-Omicron antigen booster contacts considering both wildtype-based vaccinations and/or infections with pre-Omicron variants. The patients of our cohort had a mean of 3.45 (2–5) pre-Omicron antigen contacts at this point of time. A second analysis focused on short-term effects of a first Omicron antigen booster contact by Omicron-adapted vaccination or infection with an Omicron variant (four to eight weeks after this Omicron contact). Abbreviations: IgG: immunglobulin G; IGRA: Interferon-Gamma Releasing Assay; sNABs: surrogate neutralizing antibodies.

This is the final report of our prospective, longitudinal cohort study in patients with GI cancer treated at the Department of Internal Medicine I, Gastroenterology Oncology Section at the University Hospital of Bonn, Germany. The timeline since the start of the observation period in January 2021 and design of the study are shown in Figure 4.

During the initial phase, short-term immune responses to basic SARS-CoV-2 immunization, defined as two mRNA-based vaccinations against the wildtype of SARS-CoV-2, were evaluated until April 2022 [20]. In subsequent follow-up studies, effects of a first wildtype-based booster vaccination were investigated up to July 2022 [21,22].

For the current analysis, we focused on the effect of a first Omicron antigen contact. Therefore, we initially evaluated longer-term effects of pre-Omicron booster antigen contacts (36 weeks after first pre-Omicron booster) until September 2023 (pre-Omicron era). At this point, the patients had a mean of 3.45 (2–5) pre-Omicron antigen contacts. Vaccinations with mRNA-based vaccines against the wildtype and/or infections with pre-Omicron variants were categorized as contacts with a SARS-CoV-2 antigen. For the latter, both PCR- and antigen-confirmed infections as well as silent infections with detection of SARS-CoV-2 anti-nucleocapsid IgG were considered (Table 2). Variant determinations were completed for patients with PCR-confirmed infections to exclude patients with early Omicron infections. In cases with antigen-confirmed infections, the currently locally predominant variant was assumed. The analysis included levels of SARS-CoV-2 anti-spike immunoglobulins (IgG), the capacities to neutralize wildtype and cross-reactively Omicron-variants of SARS-CoV-2, and the cellular immune response.

**Table 2:** SARS-CoV-2-associated immunological and clinical characteristics.

	Control 1		Control 2		Group 1		Group 2		Group 3	
	(*n* = 37)		(*n* = 22)		(*n* = 39)		(*n* = 26)		(*n* = 44)	
Number of vaccinations										
2	5.4%	(2)	4.5%	(1)	5.1%	(2)	3.8%	(1)	6.8%	(3)
3	43.2%	(16)	40.9%	(9)	61.5%	(24)	26.9%	(7)	43.2%	(19)
≥4	51.4%	(19)	54.6%	(12)	33.4%	(13)	69.2%	(18)	50.0%	(22)
SARS-CoV-2 anti-spike IgG ≥ 847.0 BAU/mL (pre-Omicron)										
No	37.8%	(14)	31.8%	(7)	38.5%	(15)	30.8%	(8)	36.4%	(16)
Yes	62.2%	(23)	68.2%	(15)	61.5%	(24)	69.2%	(18)	63.6%	(28)
IGRA count (pre-Omicron)										
Positive	5.4%	(2)	40.9%	(9)	33.3%	(13)	34.6%	(9)	25.0%	(11)
Negative	21.6%	(8)	4.5%	(1)	7.7%	(3)	3.8%	(1)	6.8%	(3)
Not analyzed	73.0%	(27)	54.5%	(12)	59.0%	(23)	61.5%	(16)	68.2%	(30)
Infected with a pre-Omicron variant										
No	78.4%	(29)	90.9%	(20)	89.7%	(35)	88.5%	(23)	79.5%	(35)
Yes	21.6%	(8)	9.1%	(2)	10.3%	(4)	11.5%	(3)	20.5%	(9)
Infected with an Omicron-variant										
No	70.3%	(26)	40.9%	(9)	69.2%	(27)	57.7%	(15)	54.5%	(24)
Yes	29.7%	(11)	59.1%	(13)	30.8%	(12)	42.3%	(11)	45.5%	(20)
Omicron-adapted vaccination										
No	81.1%	(30)	68.2%	(15)	100%	(39)	73.1%	(19)	84.1%	(37)
Yes	18.9%	(7)	31.8%	(7)	-		26.9%	(7)	15.9%	(7)
Clinical course of Omicron infections (WHO clinical progression Scale)										
Scale 1–3 (non-hospitalized)	100%	(11)	100%	(13)	100%	(12)	90.9%	(10)	95.0%	(19)
Scale ≥ 4 (hospitalized)	-		-		-		9.1%	(1)	5.0%	(1)

Comparison between Control 1 (patients in follow-up care without underlying liver disease), Control 2 (patients in follow-up care with underlying liver disease), Group 1 (patients with hepatocellular carcinoma), Group 2 (patients with hepatic metastatic gastrointestinal cancer), and Group 3 (patients with non-hepatic metastatic cancer). Abbreviations: IgG: immunoglobulin G; IGRA: Interferon-Gamma Releasing Assay; SARS-CoV-2: severe acute respiratory syndrome coronavirus type 2; WHO: World Health Organization.

From the beginning of the second calendar week in 2022, Omicron became the predominant variant of SARS-CoV-2 in Germany [45], which it still is today. Since October 2022, infection rates increased significantly in our cohort, for which we, therefore, assumed infections with the Omicron variant (Table 3). As most patients remained in the outpatient setting, variant determination was no longer performed. In addition, we assumed silent infections with an Omicron variant in patients who developed seroconversion for SARS-CoV-2 anti-nucleocapsid IgG for the first time from October 2022. Omicron-adapted vaccines also became available at that time. From October 2022 to September 2023, SARS-CoV-2 immune responses were re-evaluated 4 to 12 weeks after a first Omicron antigen booster via variant-adapted vaccination and/or infection (Omicron era). The period for titer measurement was extended because SARS-CoV-2 was less present in the public eye and patients were less motivated to report for additional study visits. SARS-CoV-2 anti-spike immunoglobulins (IgG), the capacities to neutralize Omicron-variants of SARS-CoV-2, and the cellular immune response were remeasured, thereby presenting short-term data on the effect of an Omicron-based booster on the long-term immune response.

**Table 3:** Baseline characteristics.

	Control 1		Control 2		Group 1		Group 2		Group 3	
	(*n* = 37)		(*n* = 22)		(*n* = 39)		(*n* = 26)		(*n* = 44)	
Age [years]										
Mean (range)	65	(33–85)	69	(44–84)	69	(49–85)	67	(48–87)	67	(45–89)
Sex										
Male	45.9%	(17)	45.5%	(10)	74.4%	(29)	61.5%	(16)	70.5%	(31)
Female	54.1%	(20)	54.5%	(12)	25.6%	(10)	38.5%	(10)	29.5%	(13)
Cancer types										
HCC	10.8%	(4)	22.7%	(5)	100%	(39)	-		-	
GEJC	18.9%	(7)	9.1%	(2)	-		-		18.2%	(8)
Esophageal cancer	2.7%	(1)	9.1%	(2)	-		-		11.4%	(5)
Gastric cancer	16.2%	(6)	-		-		-		6.8%	(3)
PBN	13.5%	(5)	13.6%	(3)	-		15.4%	(4)	34.1%	(15)
Pancreatic cancer	10.8%	(4)	-		-		7.7%	(2)	18.2%	(8)
BTC	2.7%	(1)	13.6%	(3)	-		7.7%	(2)	15.9%	(7)
CRC	27.0%	(10)	40.9%	(9)	-		38.5%	(10)	29.5%	(13)
NET	18.9%	(7)	4.5%	(1)	-		42.3%	(11)	9.1%	(4)
Other	10.8%	(4)	9.1%	(2)	-		3.8%	(1)	9.1%	(4)
Duodenal cancer	2.7%	(1)	-		-		-		6.8%	(3)
GIST	5.4%	(2)	4.5%	(1)	-		3.8%	(1)	2.3%	(1)
CUP	2.7%	(1)	4.5%	(1)	-		-		-	
Type of treatment										
Chemotherapy *	-		-		-		23.1%	(6)	31.8%	(14)
Immune checkpoint inhibitors **	-		-		7.7%	(3)	7.7%	(2)	6.8%	(3)
Targeted therapy ***	-		-		10.3%	(4)	3.8%	(1)	11.4%	(5)
Local therapy ****	-		-		41.0%	(16)	3.8%	(1)	15.9%	(7)
Combined therapy *****	-		-		38.5%	(15)	26.9%	(7)	25.0%	(11)
Other ******	-		-		2.6%	(1)	34.6%	(9)	9.1%	(4)
Immunodeficiency ^#^										
No	91.9%	(34)	95.5%	(21)	87.2%	(34)	88.5%	(23)	97.7%	(43)
Yes	8.1%	(3)	4.5%	(1)	12.8%	(5)	11.5%	(3)	2.3%	(1)
Underlying Chronic liver disease										
No	100%	(37)	-		15.4%	(6)	80.8%	(21)	77.3%	(34)
Yes	-		100%	(22)	84.6%	(33)	19.2%	(5)	22.7%	(10)
Cirrhotic	-		13.6%	(3)	78.8%	(26)	40.0%	(2)	-	
Non-cirrhotic	-		86.4%	(19)	21.2%	(7)	60.0%	(3)	100%	(10)
Etiology of liver diseases										
ASH	-		13.6%	(3)	36.4%	(12)	60.0%	(3)	30.0%	(3)
Autoimmune hepatitis	-		-		3.0%	(1)	-		-	
Budd-Chiari-Syndrome	-		4.5%	(1)	-		-		-	
Chemotherapy-induced hepatopathy	-		4.5%	(1)	-		-		-	
Post-resection liver dysfunction	-		4.5%	(1)	-		-		10.0%	(1)
Hemochromatosis	-		-		6.1%	(2)	-		-	
Hepatitis A	-		4.5%	(1)			-		-	
Hepatitis B	-		9.1%	(2)	9.1%	(3)	-		10.0%	(1)
Hepatitis C	-		9.1%	(2)	9.1%	(3)	-		10.0%	(1)
Idiopathic	-		4.5%	(1)	6.1%	(2)	-		-	
MASLD	-		40.9%	(9)	30.3%	(10)	40.0%	(2)	40.0%	(4)
PSC	-		4.5%	(1)	-		-		-	
Blood Transfusion received										
No	100%	(37)	95.5%	(21)	76.9%	(30)	92.3%	(24)	86.4%	(38)
Yes	-		4.5%	(1)	23.1%	(9)	7.7%	(2)	13.6%	(6)
Lethal outcome due to underlying cancer										
No	100%	(37)	100%	(22)	76.9%	(30)	88.5%	(23)	79.5%	(35)
Yes	-		-		23.1%	(9)	11.5%	(3)	20.5%	(9)

Comparison between Control 1 (patients in follow-up care without underlying liver disease), Control 2 (patients in follow-up care with underlying liver disease), Group 1 (patients with hepatocellular carcinoma), Group 2 (patients with hepatic metastatic gastrointestinal cancer), and Group 3 (patients with non-hepatic metastatic cancer). * Capecitabine, Cisplatin, Docetaxel, 5-Fluorouracil, Gemcitabine, Irinotecan, Oxaliplatin, Paclitaxel, Tipiracil, Trifluridine. ** Atezolizumab, Nivolumab, Pembrolizumab. *** Bevacizumab, Cabozantinib, Imatinib, Lenvatinib, Panitumumab, Sorafenib. **** Microwave Ablation, Radiotherapy, Selective Internal Radiation Therapy, surgical resection, Transarterial Chemoembolization. ***** immune checkpoint inhibitors + targeted therapy, immune checkpoint inhibitors + local therapy, immune checkpoint inhibitors + chemotherapy, chemotherapy + local therapy, chemotherapy + targeted therapy. ****** somatostatin analogues, therapies in the context of clinical studies. ^#^ Immunosuppression was assumed in patients with HIV infection and CD4^+^-cell count < 200 cells/µL and/or under therapy with Calcineurin-Inhibitors or glucocorticoids > 10 mg/day. Abbreviations: ASH: alcoholic steatohepatitis; BTC: biliary tract cancer, i.e., cholangiocellular cancer and gallbladder cancer; CRC: colorectal cancer; CUP: cancer of unknown primary; GEJC: gastroesophageal junction cancer; GIST: gastrointestinal stromal tumor; HCC: hepatocellular carcinoma; IGRA: Interferon-Gamma Release Assay; MASLD: metabolic dysfunction-associated steatotic liver disease; NET: neuroendocrine tumor; PBN: pancreaticobiliary neoplasms; PSC: primary sclerosing cholangitis.

The study was conducted in accordance with the Declaration of Helsinki, approved by the Institutional Review Board of the Medical Faculty of the University of Bonn (Nos. 341/17 and 023/22). All patients signed an informed consent form, were informed about clinically relevant results of their immunological test results and received individual advice on further vaccinations and/or strategies in the event of infection with SARS-CoV-2.

### 4.2. Patients’ Characteristics and Eligibility Criteria

All patients with active cancer underwent oncologic treatment at the time of study evaluation. Patients with HCC had previously shown particularly poor immune responses to SARS-CoV-2 antigen contacts [20,21,22,46]. In addition, liver metastases and/or underlying liver disease were considered to investigate the influence of morphological and/or functional changes in the liver on immune responses. Therefore, the following groups were defined: patients with HCC (group 1), patients with hepatic metastatic GI cancer (group 2), and patients with non-hepatic metastatic GI cancer (group 3). The latter included cases without any metastases under neoadjuvant or adjuvant therapy and cases with metastases at sites other than the liver. GI cancer other than HCC included gastroesophageal junction cancer (GEJC: gastric cancer, esophageal cancer), pancreaticobiliary neoplasms (PBN: pancreatic cancer and biliary tract cancer), colorectal cancer (CRC), neuroendocrine tumors, and other cancers (duodenal cancer, gastrointestinal stroma tumors, and cancer of unknown primary with most likely GI origin). Patients in follow-up care with a past medical history of GI cancer without active cancerous disease and not under maintenance therapy for at least 12 months prior to inclusion were involved as control groups. In these patients, a distinction was made as to whether underlying liver diseases were absent (control 1) or present (control 2).

Standardized medical records were used from the Hospital Information System Orbis (version 08044202.01000.DACHL, DH Healthcare GmbH, Bonn, Germany) to document other possible factors influencing the immune response to exposure to the SARS-CoV-2 antigen (Table 1 and Table 2). Those included the location of the cancer, types of oncological treatment (systemic therapies including chemotherapy, targeted therapy, immune-checkpoint inhibitors, as well as combined local and systemic therapies), underlying immunodeficiency (suspected in patients with long-term immunosuppressive medication and chronic HIV infection with CD4^+^-cell count < 200/µL), underlying liver diseases, history of SARS-CoV-2 infection, age, and sex. Data on the course of infections with an Omicron variant were considered using the World Health Organization (WHO) Clinical Progression Scale [47,48,49], by which patients were categorized into a non-hospitalized (score 1–3) and a hospitalized cohort (score ≥ 4) for the present analysis.

Patients under best supportive care and/or with decompensated liver function (Child-Pugh B/C score ≥ 8) who were not eligible for cancer treatment were excluded from the study. Moreover, patients who had completed their treatment within the 12 months prior to recruitment were not considered. For the analysis during the Omicron era, those patients without any additional Omicron antigen exposure were excluded.

### 4.3. Assessment of Humoral and Cellular Response Rates

Quantification of SARS-CoV-2 anti-spike IgG levels, as well as of the capacity to neutralize the wildtype virus and analysis of the cellular immune response, was performed as previously described [20,21,22]. The capacity to neutralize the Omicron variants of SARS-CoV-2 was determined using cPassTM SARS-CoV-2 Neutralization Antibody Detection Kit Omicron Variant (GenScript). This enzyme-linked immunosorbent assay measures surrogate neutralizing antibodies (sNABs), and thus, detects functional effects of antibodies, which are strongly associated with live-virus neutralization of the Omicron variants [50,51].

For the present study, we assumed protection against severe courses of COVID-19 at a titer of 847.0 BAU/mL or higher. The value of 847.0 BAU/mL was above the 20th percentile in immunocompetent patients in a seroprevalence study, while values below the 20th percentile were associated with a higher mortality rate for breakthrough infections [52,53].

### 4.4. Statistical Analysis

Statistical analysis was carried out using R version 4.3.3 (R Core Team 2024: R: a language and environment for statistical computing, R Foundation for Statistical Computing, Vienna, Austria). Descriptive analyses included the calculation of medians and interquartile ranges for continuous variables and frequencies (absolute and relative) for categorical variables.

Univariate linear mixed effects models were used to compare (log_10_ transformed) levels of SARS-CoV-2 anti-spike IgG as well as neutralization of the wildtype and the Omicron variants in the pre-Omicron era and in the Omicron era across the five groups and with respect to cancer type and type of treatment, respectively. Each model contains main effects, interaction terms with time, as well as a patient-specific random intercept. Additionally, a univariate logistic mixed effects model was used for modeling the probabilities of achieving an effective titer (SARS-CoV-2 anti-spike IgG of 847.0 BAU/mL) for the five groups at the two points in time. Neutralization of the wildtype and the Omicron variants were compared in the pre-Omicron era applying a simple linear model. Furthermore, multivariable regression analyses (for SARS-CoV-2 anti-spike IgG and the capacities to neutralize the wildtype and the Omicron variants) were performed to examine a possible effect of age, sex, history of SARS-CoV-2 infection, and additional immunosuppression. In this study, *p*-values ≤ 0.05 were regarded as statistically significant.

## 5. Conclusions

Clinical courses of a first infection with an Omicron variant of SARS-CoV-2 were predominantly mild to moderate in our cohort of patients with active GI cancer. This suggests that pre-Omicron immunological protection after at least three SARS-CoV-2 antigen contacts is also effective against these emerging variants of SARS-CoV-2. From a clinical point of view, pre-Omicron immunological protection after at least three SARS-CoV-2 antigen contacts was also effective in the case of a first infection with an Omicron variant in our cohort of patients with active GI cancer. However, we demonstrated that booster antigen contacts can only temporarily stabilize waning humoral immune responses and that initial contacts with SARS-CoV-2 antigens of emerging variants continues to trigger only reduced immune responses. Vaccination gaps should, thus, be closed urgently. Our study underscores the importance of continued monitoring and tailored vaccination strategies for GI cancer patients to ensure sustained protection against evolving SARS-CoV-2 variants. Based on the presented data, among patients with GI carcinoma, especially patients with PBN, patients undergoing conventional chemotherapy and patients with systemic immunodeficiency show poorer responses to SARS-CoV-2 antigen contacts. For the time being, booster vaccinations should be recommended in line with the national and international guidelines to maintain long-term protection against SARS-CoV-2.

## Figures and Tables

**Table 1:** Titers of SARS-CoV-2 anti-spike IgG and wildtype- and Omicron-neutralization.

	SARS-CoV-2 Anti-Spike IgG [log_10_ BAU/mL]						Wildtype sNAB [%]			Omicron sNAB [%]					
Timepoint	Pre-Omicron (1)			Omicron (2)			Pre-Omicron (1)			Pre-Omicron (1)			Omicron (2)		
	Estimate	95% CI	*p*	Estimate	95% CI	*p*	Estimate	95% CI	*p*	Estimate	95% CI	*p*	Estimate	95% CI	*p*
(a) Group comparison (intercept: liver-healthy control group)															
Control 1 (Ref.)	3.24	3.02–3.46		3.96	3.58–4.34	<0.01	93.24	87.86–98.63		71.48	62.78–80.18		93.86	78.21–100.0	<0.01
Control 2	3.26	2.97–3.55	0.91	3.52	3.18–3.86	0.09	97.52	90.14–100.0	0.36	68.00	55.60–80.41	0.65	87.41	73.39–100.0	0.80
Group 1	3.17	2.96–3.38	0.64	3.50	3.13–3.88	0.17	95.57	90.35–100.0	0.54	64.78	56.31–73.25	0.28	79.10	63.51–94.68	0.50
Group 2	3.22	2.97–3.48	0.93	3.49	3.10–3.89	0.12	95.47	88.98–100.0	0.60	76.75	66.45–87.05	0.44	90.15	73.63–100.0	0.47
Group 3	3.16	2.96–3.36	0.61	3.26	2.97–3.55	0.01	90.08	84.61–95.55	0.42	63.75	55.39–72.11	0.21	70.70	58.27–83.12	0.16
(b) Group comparison (intercept: control group with underlying liver disease)															
Control 2 (Ref.)	3.26	2.95–3.57		3.52	3.16–3.88	0.18	97.52	89.98–100.0		67.84	55.12–80.55		87.93	73.72–100.0	0.01
Group 1	3.17	2.94–3.39	0.64	3.50	3.10–3.89	0.81	95.57	90.24–100.0	0.68	64.81	56.08–73.54	0.70	79.26	63.82–94.70	0.61
Group 2	3.22	2.95–3.49	0.87	3.49	3.07–3.90	1.00	95.47	88.84–100.0	0.69	76.83	66.22–87.43	0.29	88.63	72.18–100.0	0.48
Group 3	3.16	2.94–3.38	0.62	3.26	2.95–3.57	0.51	90.08	84.49–95.67	0.12	63.76	55.14–72.38	0.60	70.92	58.52–83.33	0.20
(c) Cancer type															
No cancerous activity >1 year (Ref.)	3.24	3.07–3.42		3.71	3.46–3.96	<0.01	94.73	90.47–98.99		70.29	63.13–77.45		90.96	80.69–100.0	<0.01
HCC	3.17	2.96–3.37	0.57	3.50	3.13–3.87	0.57	95.57	90.46–100.0	0.80	64.80	56.27–73.34	0.33	79.21	63.93–94.50	0.52
GEJC	2.90	2.41–3.38	0.19	3.43	2.82–4.03	0.85	74.30	59.40–89.20	0.01	66.98	45.74–88.23	0.77	100.0	74.15–100.0	0.35
PBN	3.52	3.21–3.83	0.12	3.34	2.89–3.79	0.02	98.93	90.67–100.0	0.37	71.89	59.00–84.78	0.83	64.68	46.03–83.33	0.02
CRC	3.05	2.78–3.33	0.25	3.46	3.02–3.90	0.81	93.47	86.45–100.0	0.76	68.32	57.47–79.17	0.76	81.40	63.14–99.67	0.49
NET	3.29	2.96–3.62	0.82	3.35	2.86–3.83	0.15	93.23	84.97–100.0	0.75	73.55	59.64–87.46	0.68	85.38	65.37–100.0	0.46
Other *	2.71	2.14–3.28	0.08	2.64	1.80–3.48	0.24	82.99	69.66–96.31	0.10	50.83	24.81–76.84	0.16	45.62	10.70–80.55	0.18
(d) Type of treatment															
Off treatment > 1 year (Ref.)	3.24	3.07–3.42		3.71	3.45–3.97	<0.01	94.73	90.22–99.24		70.34	63.09–77.59		90.31	79.69–100.0	<0.01
Chemotherapy	3.25	2.95–3.55	0.99	3.10	2.56–3.64	0.05	94.93	86.78–100.0	0.97	67.22	55.21–79.24	0.66	60.52	37.90–83.14	0.05
Immune Checkpoint inhibitors	3.09	2.62–3.55	0.53	3.87	3.01–4.72	0.49	98.34	84.22–100.0	0.63	63.74	45.17–82.30	0.51	93.64	58.11–100.0	0.62
Targeted therapy	3.19	2.77–3.60	0.80	3.45	2.75–4.16	0.61	96.91	86.93–100.0	0.69	64.18	46.67–81.68	0.52	73.57	44.41–100.0	0.53
Local therapy	3.16	2.88–3.43	0.60	3.65	3.22–4.10	0.91	90.56	83.50–97.62	0.33	64.54	52.79–76.28	0.41	91.96	74.03–100.0	0.51
Combined therapy	3.07	2.84–3.31	0.25	3.13	2.76–3.50	0.09	91.97	85.78–98.16	0.48	65.41	56.00–74.83	0.41	67.19	51.20–83.19	0.09

The results of linear mixed model analysis for the SARS-CoV-2 anti-spike IgG and neutralization antibodies for wildtype SARS-CoV-2 and the Omicron variants are shown. Results are reported by mean estimates, 95% confidence intervals, and associated *p*-values. Here, *p*-values refer to the comparison to the shown reference/intercept. Control 1 (patients in follow-up care without underlying liver disease), Control 2 (patients in follow-up care with underlying liver disease), Group 1 (patients with hepatocellular carcinoma), Group 2 (patients with hepatic metastatic gastrointestinal cancer), and Group 3 (patients with non-hepatic metastatic cancer). * duodenal cancer, gastrointestinal stromal tumor, cancer of unknown primary. Abbreviations: CI: confidence interval; HCC: hepatocellular carcinoma; GI: gastrointestinal cancer; GEJN: gastroesophageal junction cancer; IgG: immunoglobulin G; PBN: pancreaticobiliary neoplasms; CRC: colorectal cancer; NET: neuroendocrine tumor; ref: reference; sNAB: surrogate neutralizing antibody.

## Data Availability

All data generated or analyzed during this study are included in this article. Further inquiries can be directed to the corresponding author.
